# Supplementary material for: Hydrogen production from the air
Source: Nat Commun. 2022 Sep 6;13:5046. doi: 10.1038/s41467-022-32652-y (PMC9448774; doi:10.1038/s41467-022-32652-y)
Supplement: Supplementary file 1 — Supplementary Information [file 41467_2022_32652_MOESM1_ESM.pdf]

Supplementary Information for

Hydrogen Production from the Air

Jining Guo[1], Yuecheng Zhang[1], Ali Zavabeti[1], Kaifei Chen[1], Yalou Guo[1], Guoping Hu[1]✉, Xiaolei Fan[2]✉ and Gang Kevin Li[1]✉

1.Department of Chemical Engineering, the University of Melbourne, Parkville, Victoria 3010, Australia.

2.Department of Chemical Engineering, School of Engineering, The University of Manchester, Manchester, M13 9PL, United Kingdom

✉email: li.g@unimelb.edu.au, xiaolei.fan@manchester.ac.uk, guoping.hu@unimelb.edu.au

List of Supplementary Information

1. Global water risk, solar potential, and wind potential.
2. Setup of Direct Air Electrolysis (DAE) module
3. Design of the Foam
4. Anode gas production using CH_3COOK electrolyte
5. Design of electrodes
6. iR-corrected J-V curves for DAE module
7. Conductivity of electrolyte
8. Viscosity of electrolyte
9. Performances and series resistances with KOH electrolyte
10. Stability of DAE
11. Design and test of prototype H_2 generation tower

12. Gas production at the cathode

13. O_2 by-production

14. Gas production at the anode

15. DAE module operating condition with triple junction solar cell

16. DAE module driven by wind turbine

17. Supplementary tables

18. Supplementary note 1

19. Supplementary reference

1. Global water risk, solar potential, and wind power potential

The Fig.1a was superimposed from Supplementary Fig.1 and Supplementary Fig.2, while the Fig.1b was superimposed from Supplementary Fig.1 and Supplementary Fig.3. The legends of the supporting figure were adjusted by Geographic Information System (GIS) software ArcGIS.

Global water risk

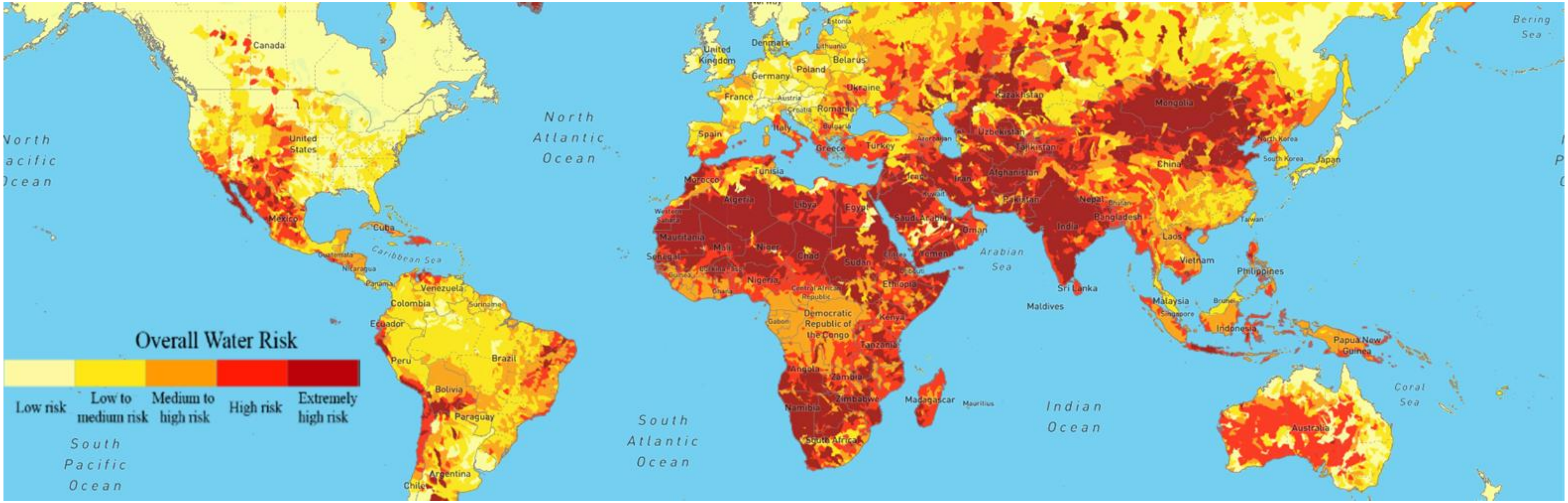

Supplementary Fig.1 Water risk all over the world. Source: World Resources Institute (WRI) Aqueduct[1], accessed on [04.2021], aqueduct.wri.org — Creative Commons Attribution International 4.0 License.

Global solar potential

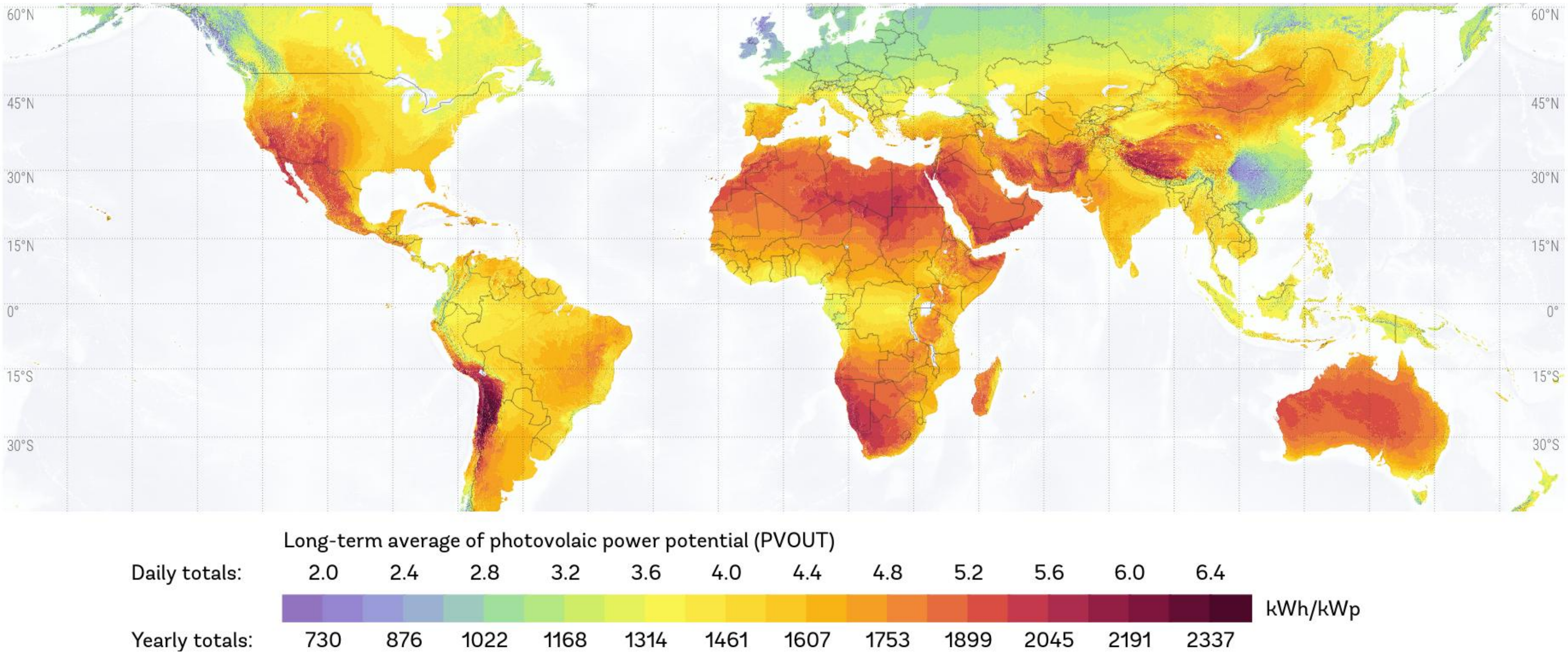

Supplementary Fig.2 Solar power potential all over the world. Source: World Bank Group[2], accessed on [04.2021], https://globalsolaratlas.info — Creative Commons Attribution International 4.0 License.

Map obtained from the "Global Solar Atlas 2.0, a free, web-based application is developed and operated by the company Solargis s.r.o. on behalf of the World Bank Group, utilizing Solargis data, with funding provided by the Energy Sector Management Assistance Program (ESMAP)."

Global wind power potential

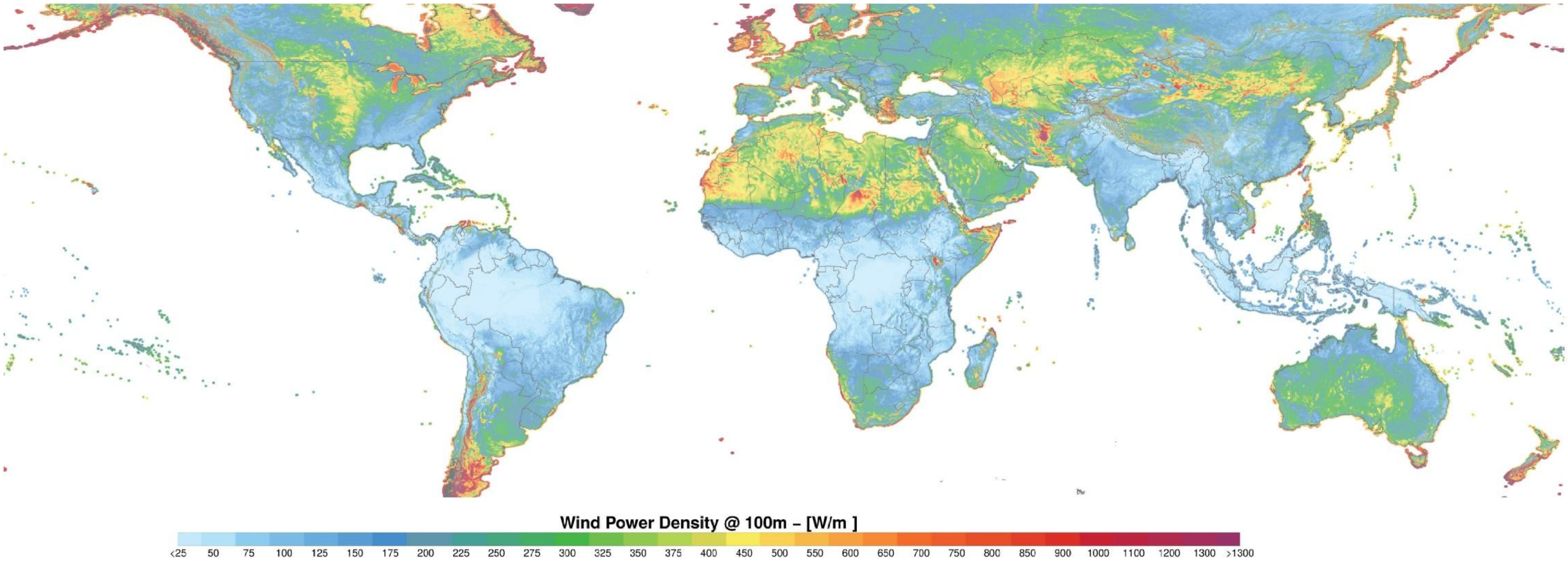

Supplementary Fig.3 Wind power density potential all over the world. Source: Technical University of Denmark[3], accessed on [04.2021], https://globalwindatlas.info — Creative Commons Attribution International 4.0 License.

Map obtained from the "Global Wind Atlas 3.0, a free, web-based application developed, owned and operated by the Technical University of Denmark (DTU). The Global Wind Atlas 3.0 is released in partnership with the World Bank Group, utilizing data provided by Vortex, using funding provided by the Energy Sector Management Assistance Program (ESMAP)."

2. Setup of Direct Air Electrolysis (DAE) module

Extra notch was designed to store the excess solution in the humid day. When the R.H. decreased, the volume of the H_2SO_4 was also decreased. However, due to capillarity, the excessed solution which stored in the notch can flow into the foam.

The thickness of the foam was the same as the distance between current collectors, shown directly in Fig. S2. However, there were two middle plates, and two sealing was used between the current collectors. Since we are now using acrylic as the material for the mid-plate, if it is too thin (<3 mm), it would be broken when assembling the module. Also, we do not want the extra liquid to flow out of the module at the anode side; the thickness was around 6 mm to ensure that the extra liquid absorbed from the air would keep in the module. The sealing we used was 0.5 mm, 1 mm separately. Also, the thickness of the nut's head should also be considered, which was 2 mm. Hence, the foam's thickness must be over (1+0.5+3+6+2*2=) 14.5 mm. The foam's thickness could be reduced if we use other materials to replace the acrylic (like some metal).

Both cathode and anode were separated from the air. Some extra ionic solution was stored between the end plate and the porous foam (Supplementary Fig.5), which meant the anode electrode was soaked in the solution. The ionic solution made sure that the anode was separate from the air. The O_2 was generated as the bubble in the ionic solution, but it could release directly into the air, shown in Supplementary Fig.5.

a

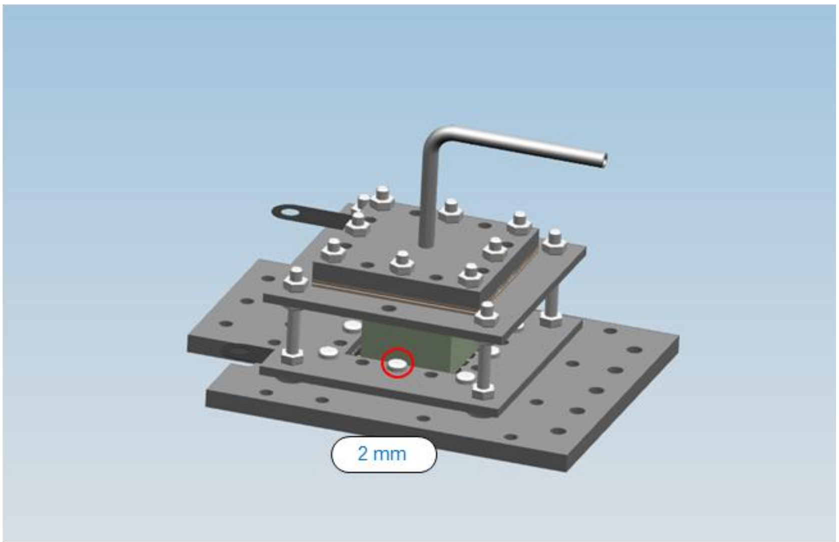

b

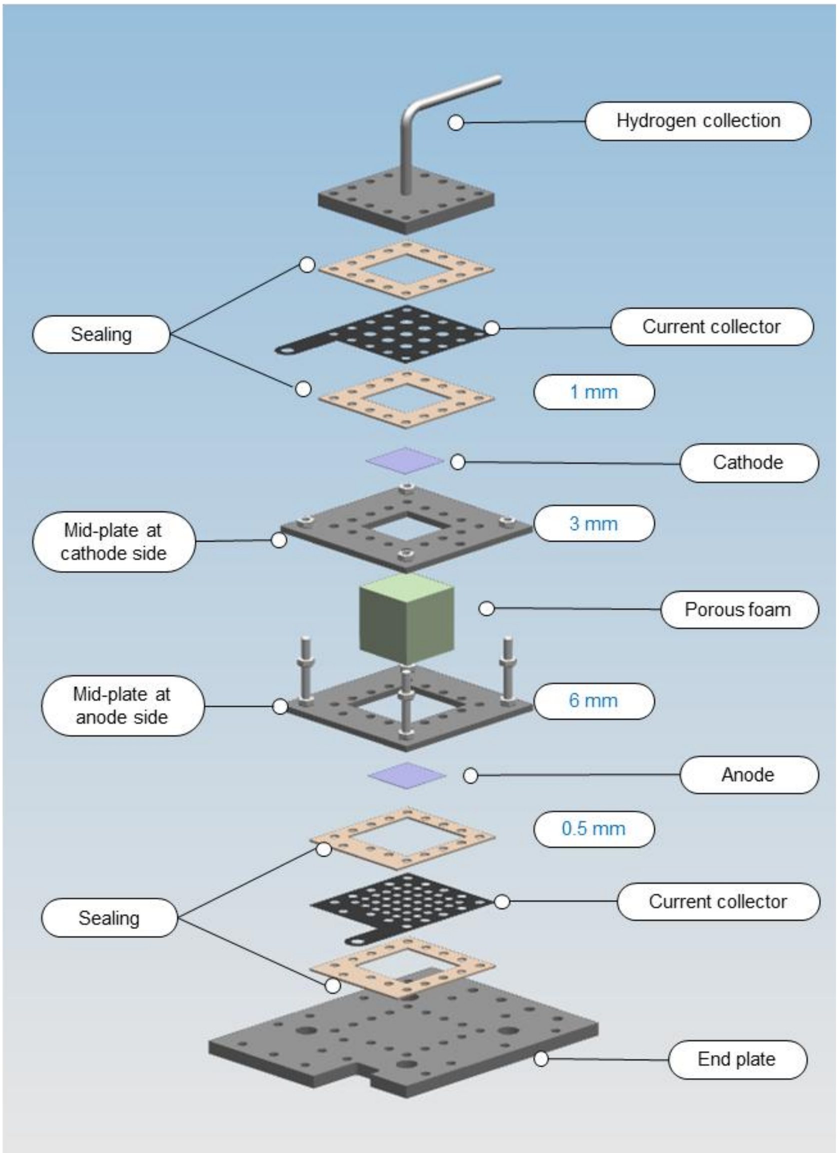

Supplementary Fig.4 Diagram for the DAE module. a Overlook of DAE module, **b** Details of DAE module.

a

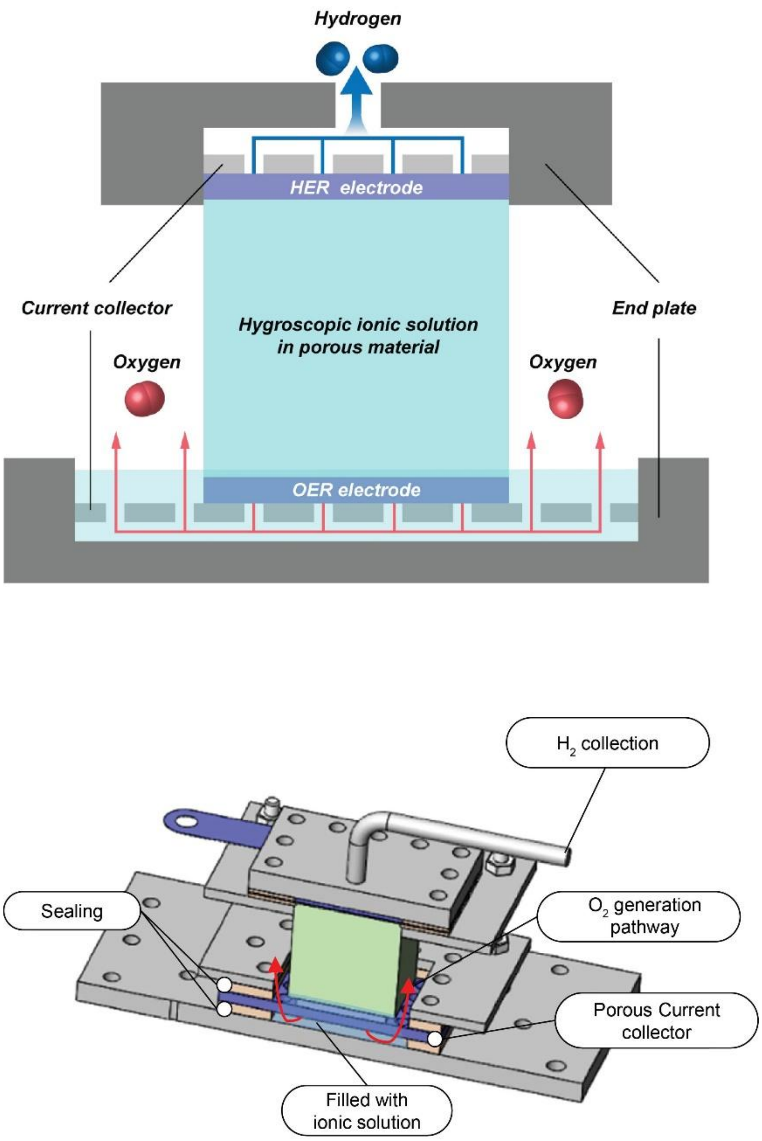

Supplementary Fig.5 Diagram for Sectional view the DAE module. a 2D, **and b** 3D view**.**

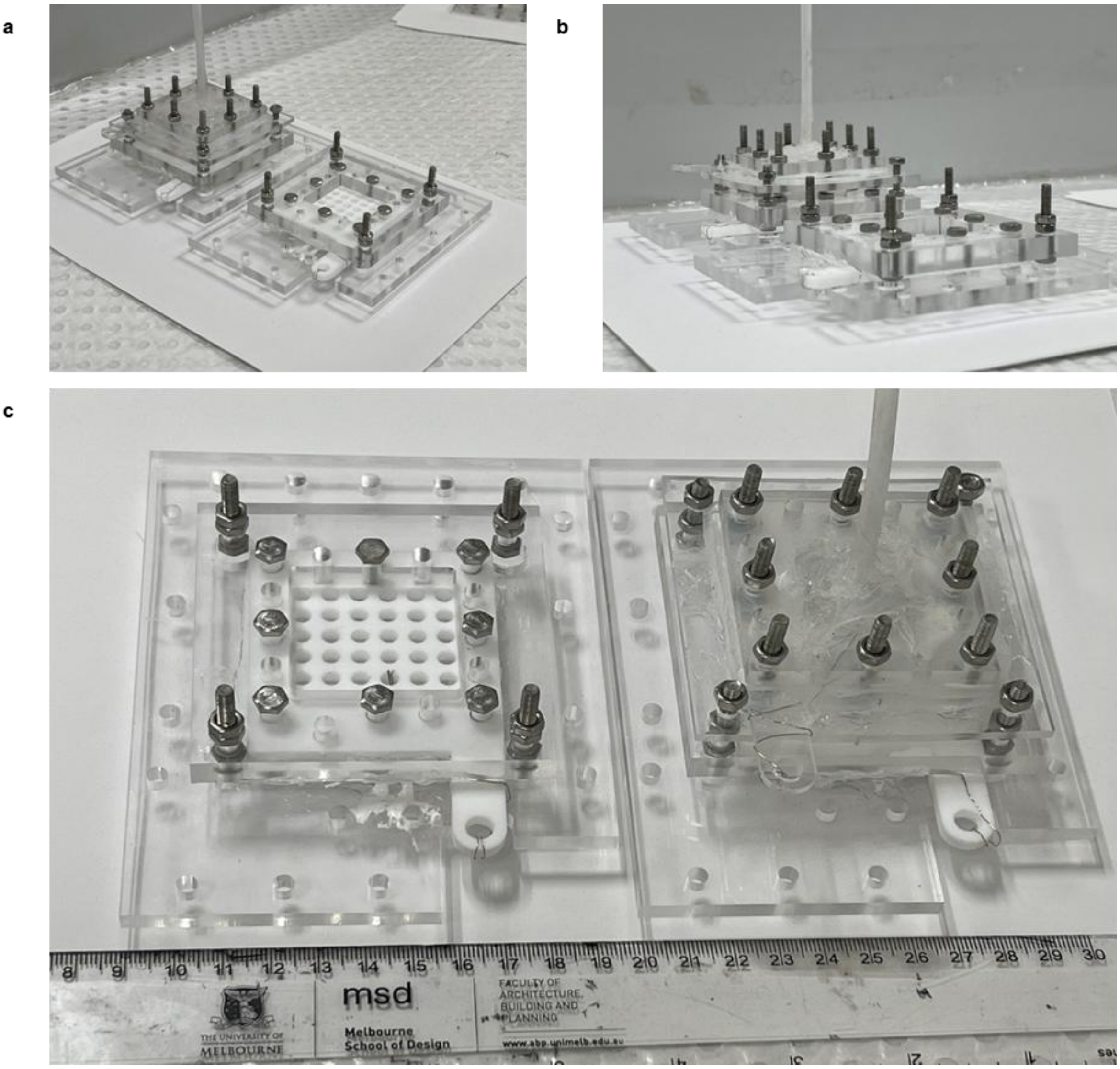

Supplementary Fig.6 Photos for the DAE module. a, b and c, details of the DAE module.

The current collector here is the PTFE plate combined with the Pt wire. Gold or Platinum coated metal plate can also be used as the current collector.

3. Design of the Foam

Sintered glass foam, which is manufactured by fusing or sintering glass particles into a solid and keeps its porous body, is chosen in this project to hold the hygroscopic electrolyte. The side surface of the form is available for moisture capture from the air.

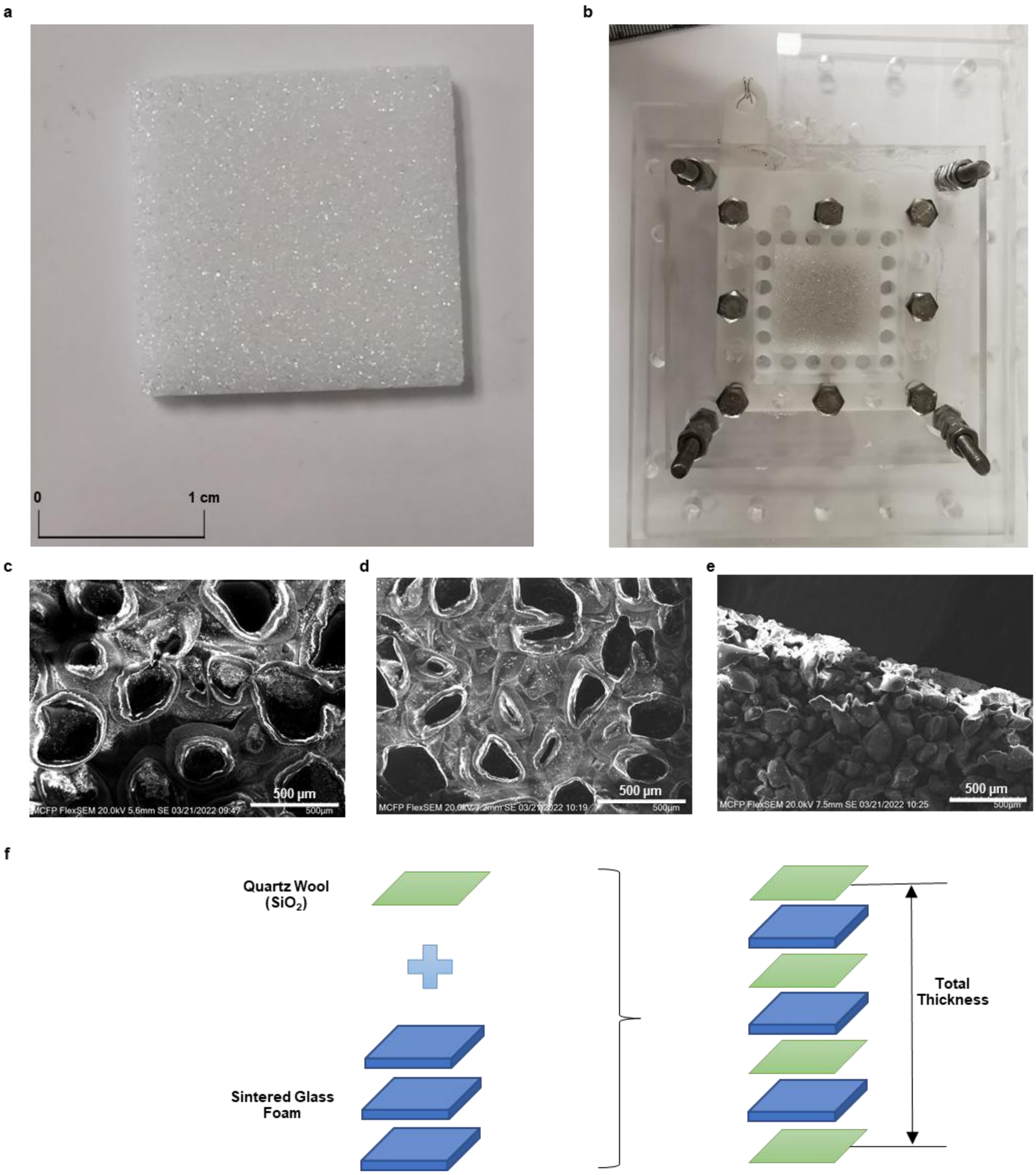

Supplementary Fig.7 Details of the sintered glass foam. a Outlook of G1 sintered glass foam; **b** Overlook when G1 sintered glass foam put in the DAE module. SEM images for **c** G1, **d** G2, **e** G3 glass foam. **f** Total thickness for sintered glass foam and quartz

wool.

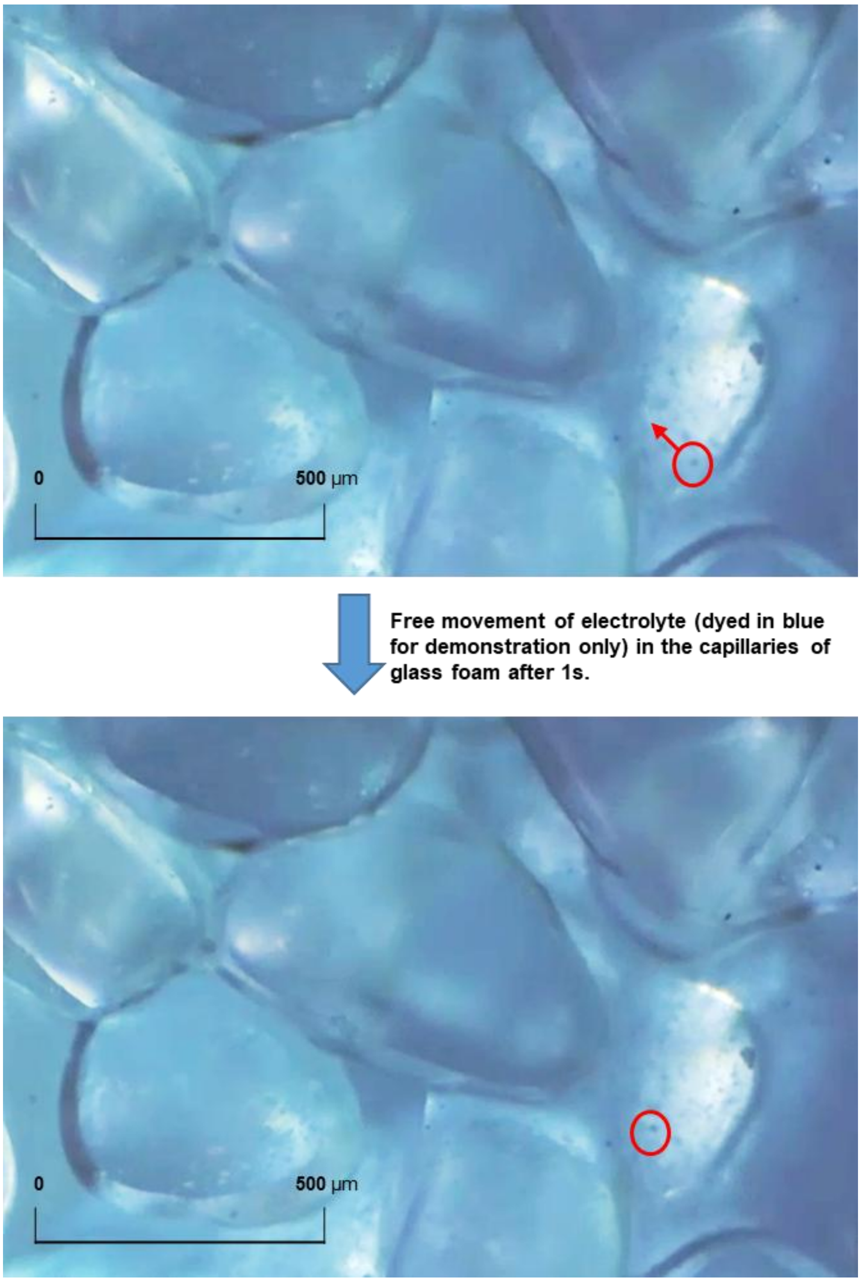

Supplementary Fig.8 Optical micro images of the free movement of dyed electrolyte in the capillaries of the G1 foam. Blue pigment was used for demonstration purpose only. See Supplementary Movie 1 for more of the motions of the electrolyte.

4. Anode gas production using CH_3COOK electrolyte

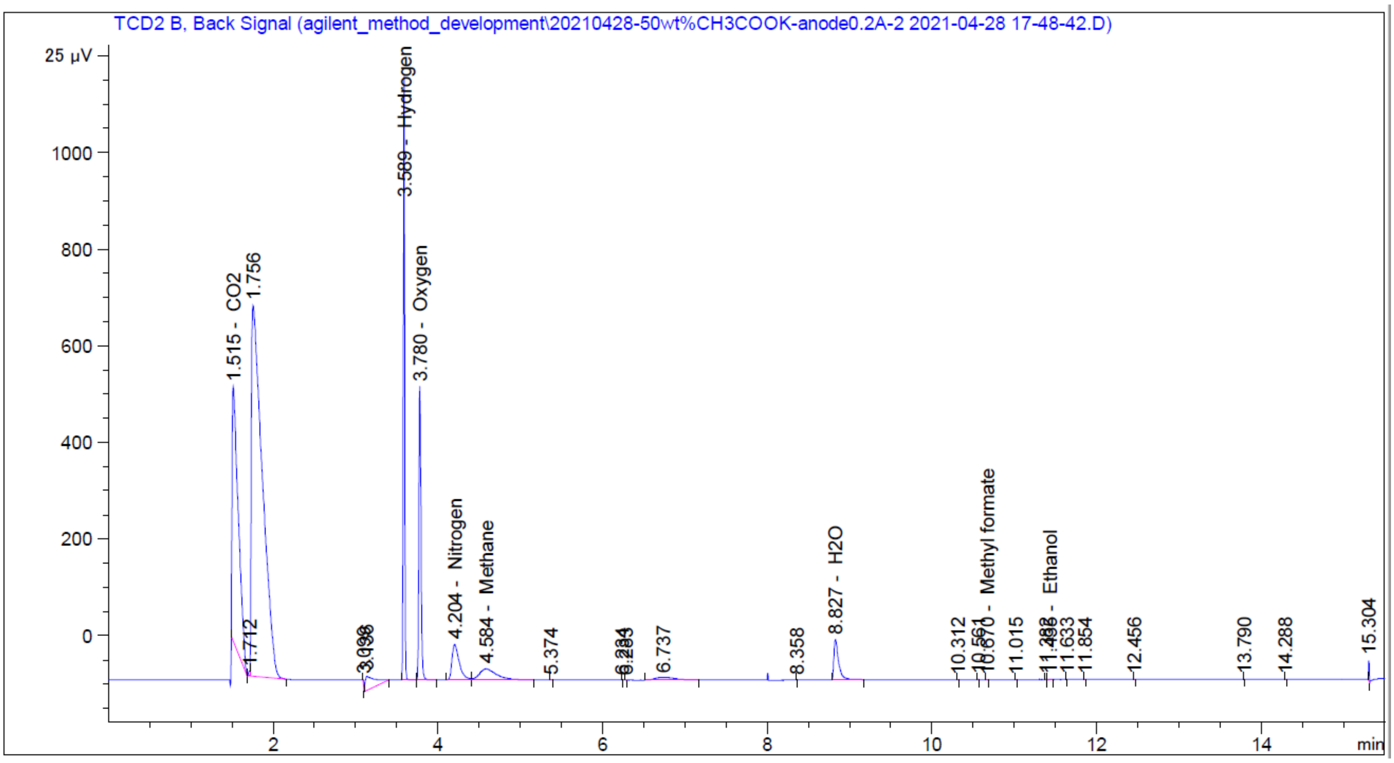

Supplementary Fig.9 Composition of the gas production at the anode by electrolyzing 50 wt% CH_3COOK checked by gas chromatography (GC.). Substantial carbon dioxide (1.515 min), ethane (1.756 min), hydrogen (3.589 min) and methane (4.584 min) were observed with oxygen (3.780 min). CH_3COOK is not the suitable choice.

5. Design of electrodes

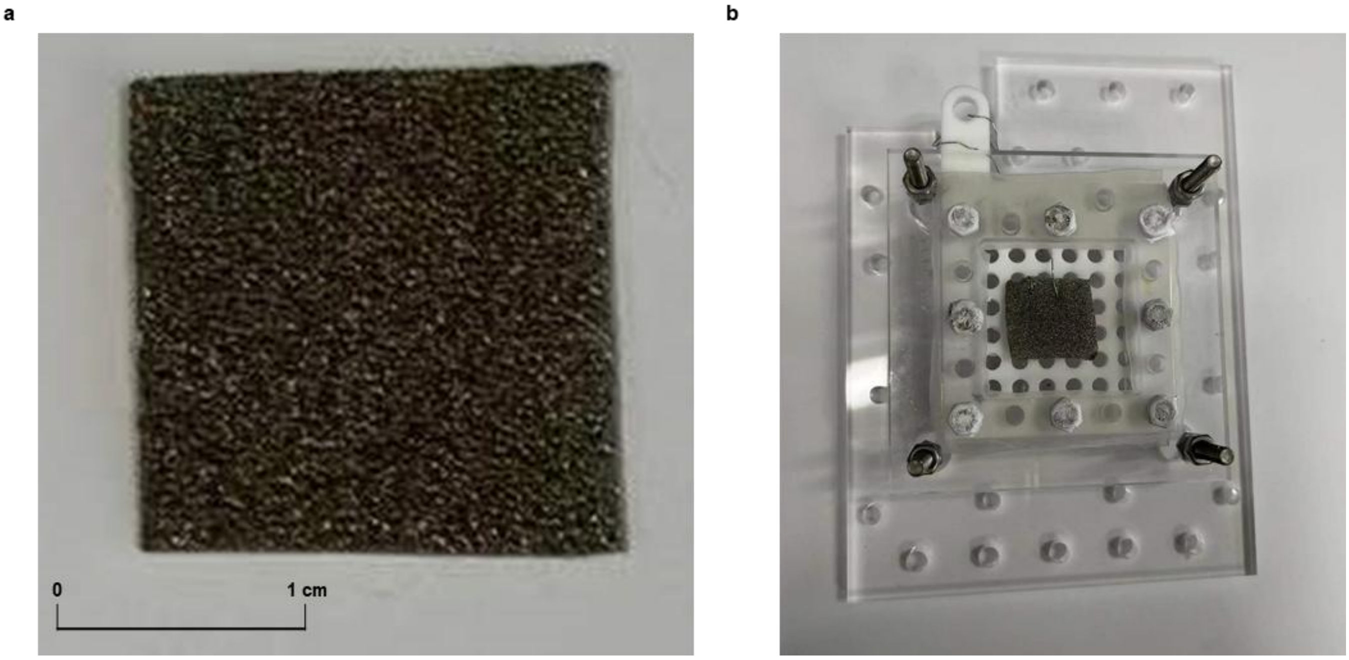

Supplementary Fig.10 Photos of the Nickel foam. a Outlook of Ni foam. **b** Overlook when Ni foam put in the DAE.

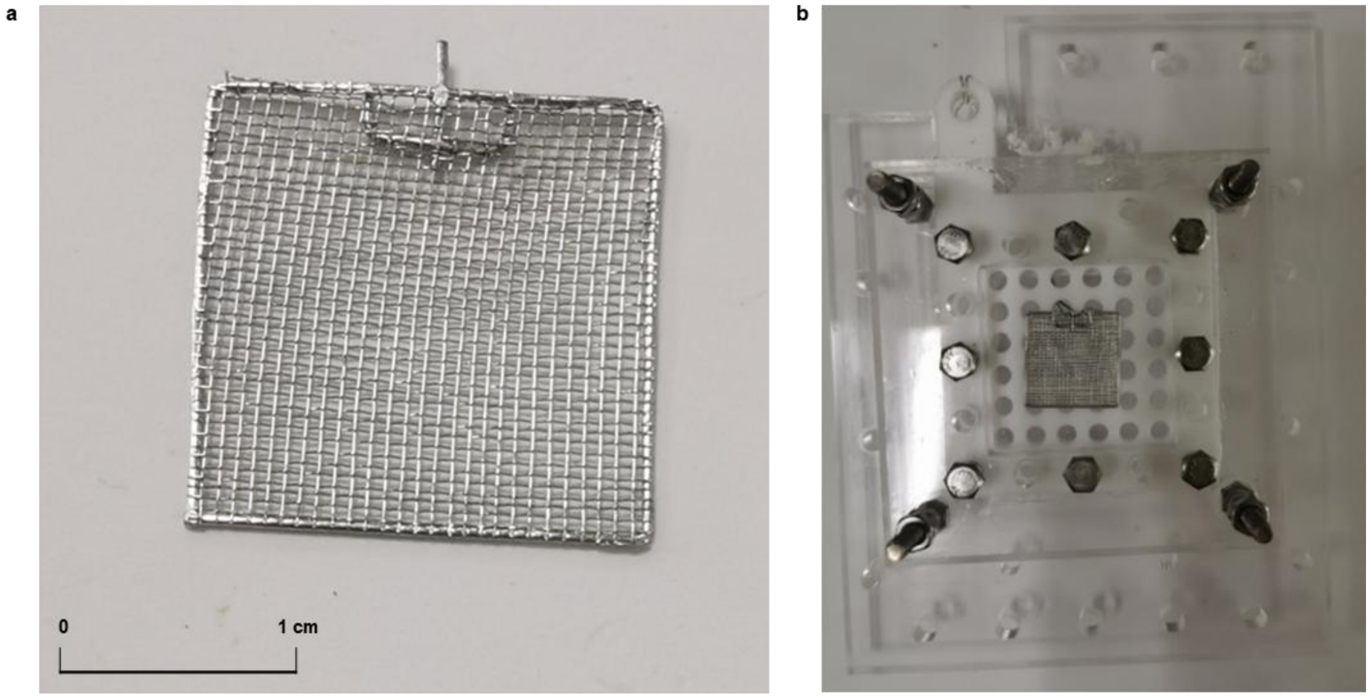

Supplementary Fig.11 Photos of the Pt net. **a** Outlook of Pt net. **b** Overlook when Pt net put in the DAE.

6. iR-corrected J-V curves for DAE module

The iR-corrected J-V curves for DAE module can reach 37.5 mA cm^{-2} at 2.75V using 62.5 wt% H_2SO_4 electrolyte but 97.0 mA cm^{-2} at 2.53V under 33.0 wt% H_2SO_4.

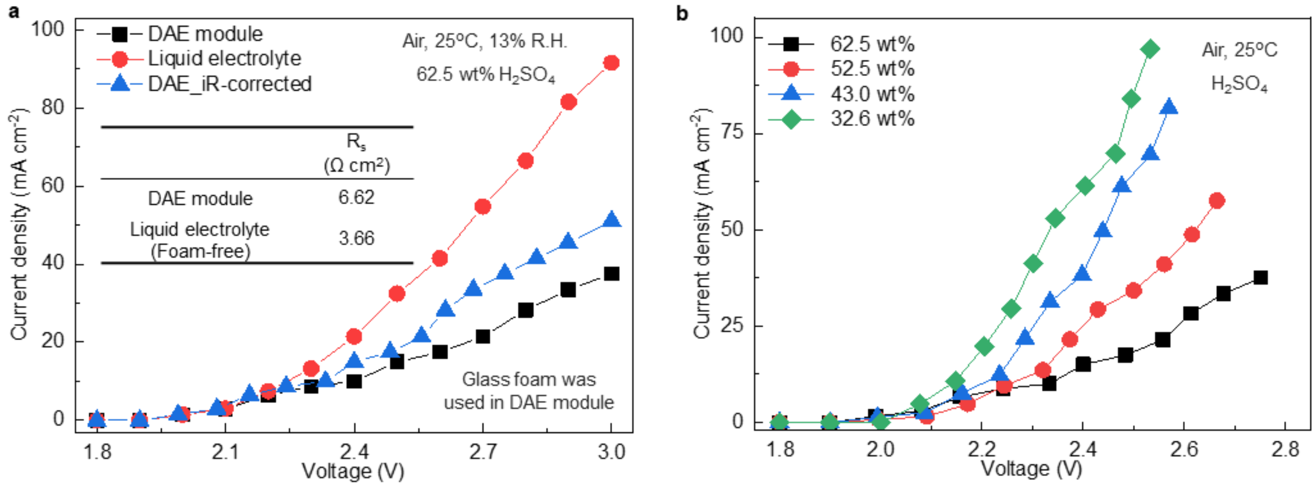

Supplementary Fig.12 iR-corrected J-V curves for DAE module. a J–V curves with 62.5 wt% H_2SO_4 (in equilibrium with 13% R.H. at 25 °C) for modules under liquid electrolyte, DAE module and iR-corrected J–V curves for DAE module. **b** iR-corrected J–V curves for DAE module under different H_2SO_4 concentration. Source data are provided as a Source Data file.

7. Conductivity of electrolyte

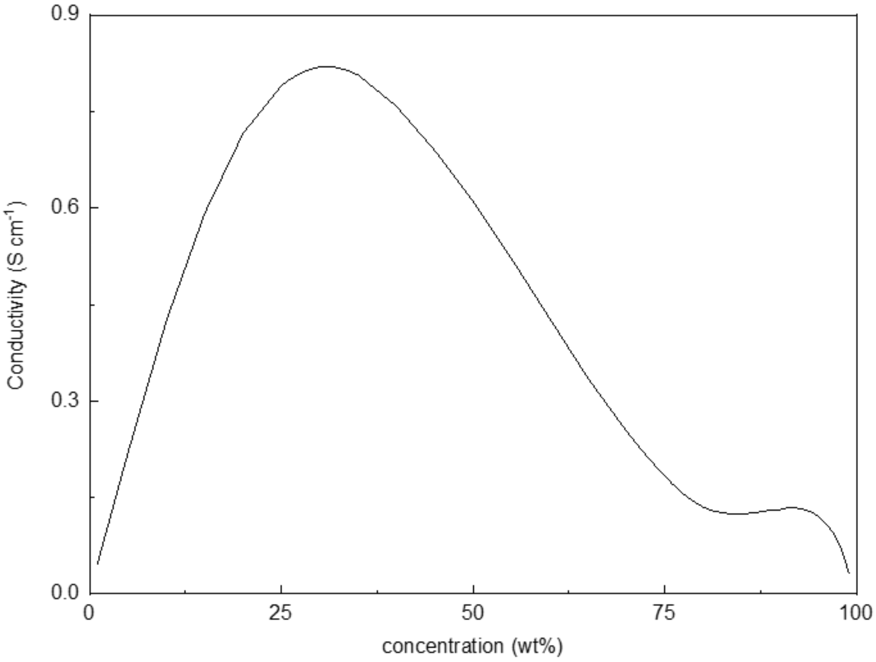

Supplementary Fig.13 Conductivity (S cm^{-1}) vs concentration (wt%) of sulfuric acid at 25°C[4,5]. Source data are provided as a Source Data file.

8. Viscosity of electrolyte

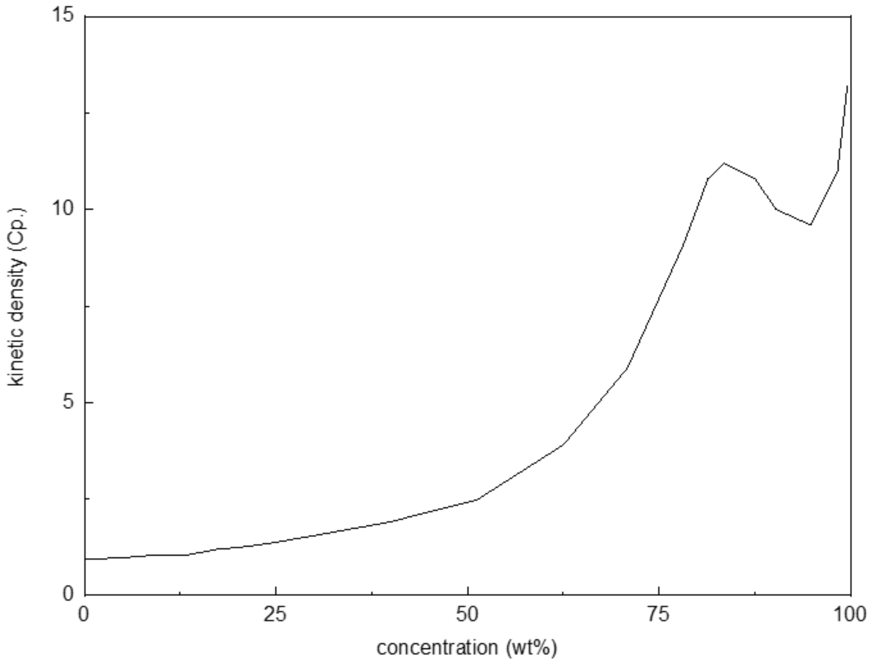

Supplementary Fig.14 Kinematic viscosity (Cp.) vs concentration (wt%) of sulfuric acid at 25°C[6]. Source data are provided as a Source Data file.

9. Performances and series resistances with KOH electrolyte

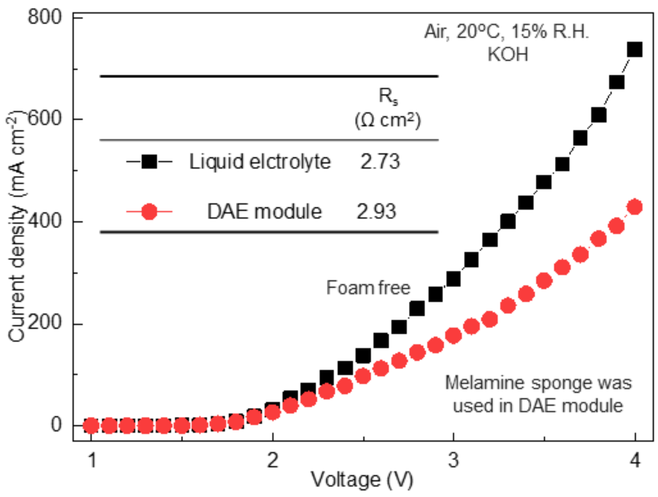

Supplementary Fig.15 J–V curves and series resistances with KOH electrolyte (in equilibrium with 15% at 20 °C) for DAE module using melamine sponge and foam-free electrolyzer. Source data are provided as a Source Data file.

10. Stability of DAE

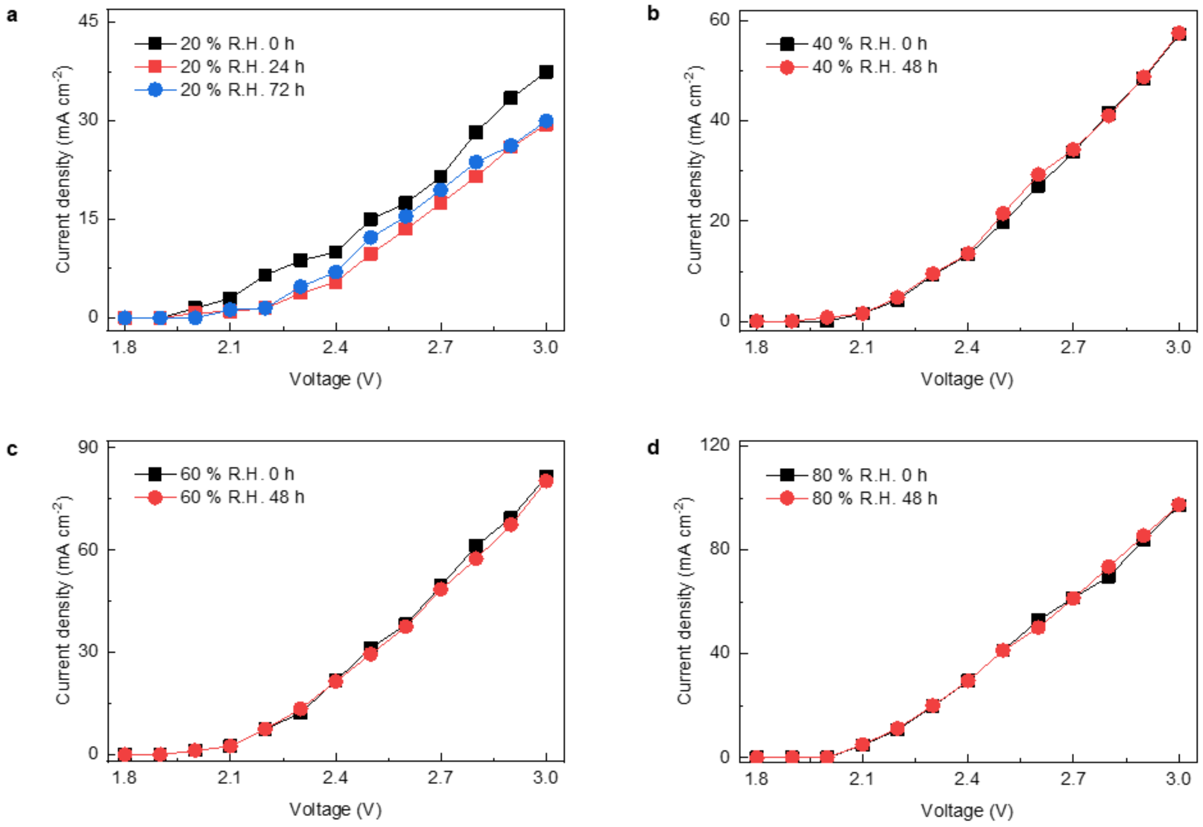

Supplementary Fig.16 J-V behavior for DAE modules operating over 48h. a R.H.= 20%; **b** R.H. = 40%; **c** R.H. = 60%; **d** R.H. = 80%. Source data are provided as a Source Data file.

11. Design and test of prototype H_2 generation tower

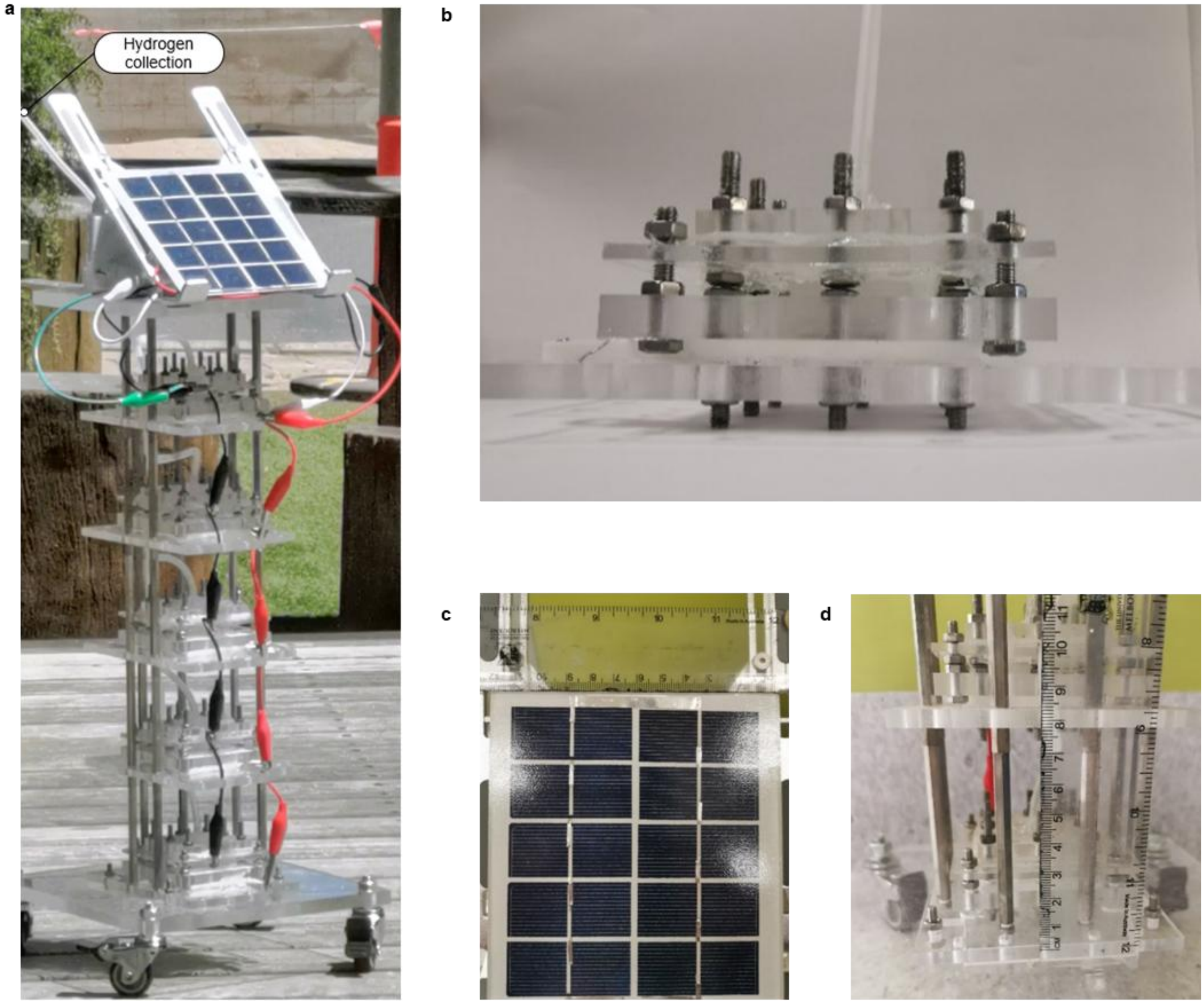

Supplementary Fig.17 Photos of the prototype H_2 generation tower. a A photo of the hydrogen generation tower. **b** Main view of the DAE module; **c** A picture of the solar panel; **d** Distance between two DAE modules.

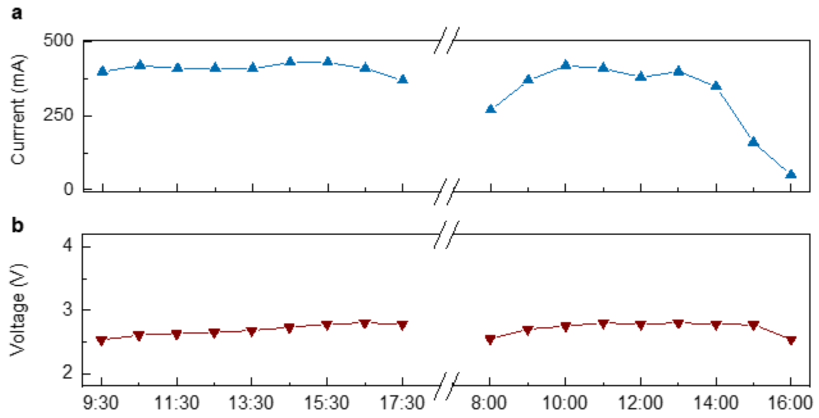

Supplementary Fig.18 Open-air demonstration of the hydrogen generation tower. a A recording of current (mA) for hydrogen generation tower each hour. **b** A recording of voltage(V) for hydrogen generation tower. Source data are provided as a Source Data file.

12. Gas product at the cathode

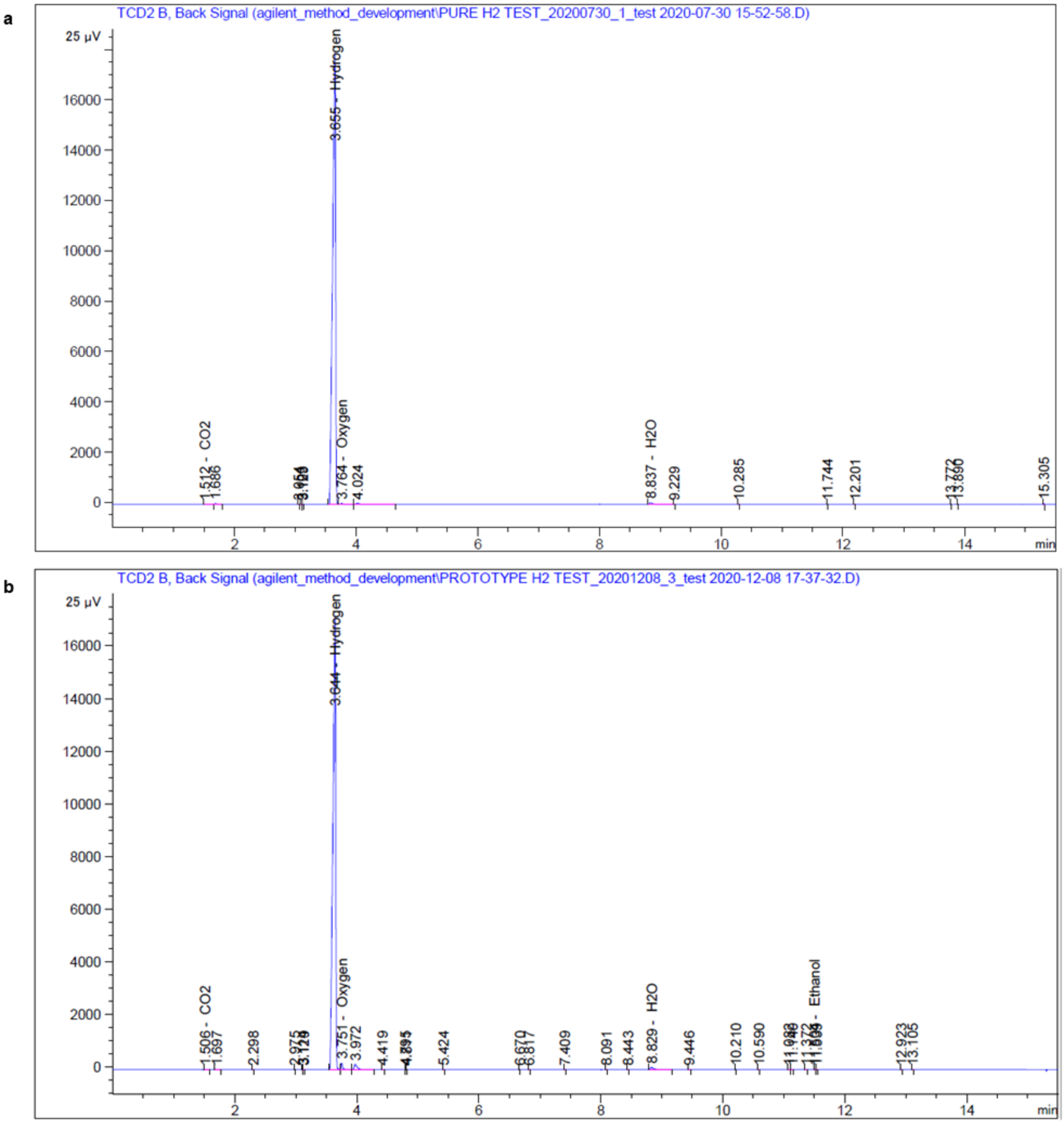

Supplementary Fig.19 Gas product from the cathode. a Composition of the gas production at the cathode by electrolyzing H_2SO_4 solution checked by gas chromatography (GC.). **b** Pure H_2 checked by GC.

Compared with Supplementary Fig.19a and Fig.19b, we can confirm that the gas production at the cathode is high purity H_2(>99%).

13. O_2 by-production

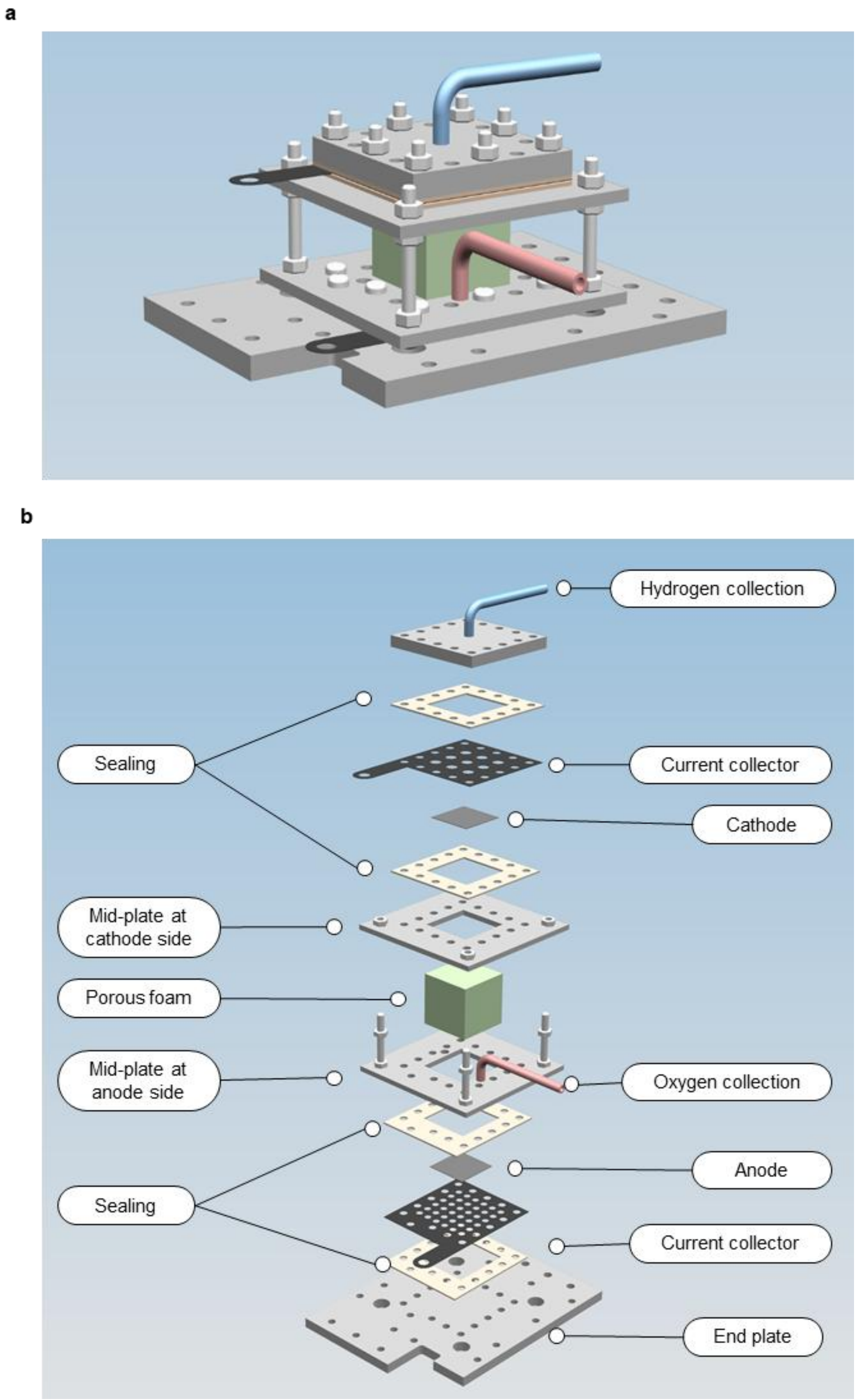

Supplementary Fig.20 Diagram for the DAE module for collecting anode gas production. a. Overlook, **b.** Details.

14. Gas product at the anode

a

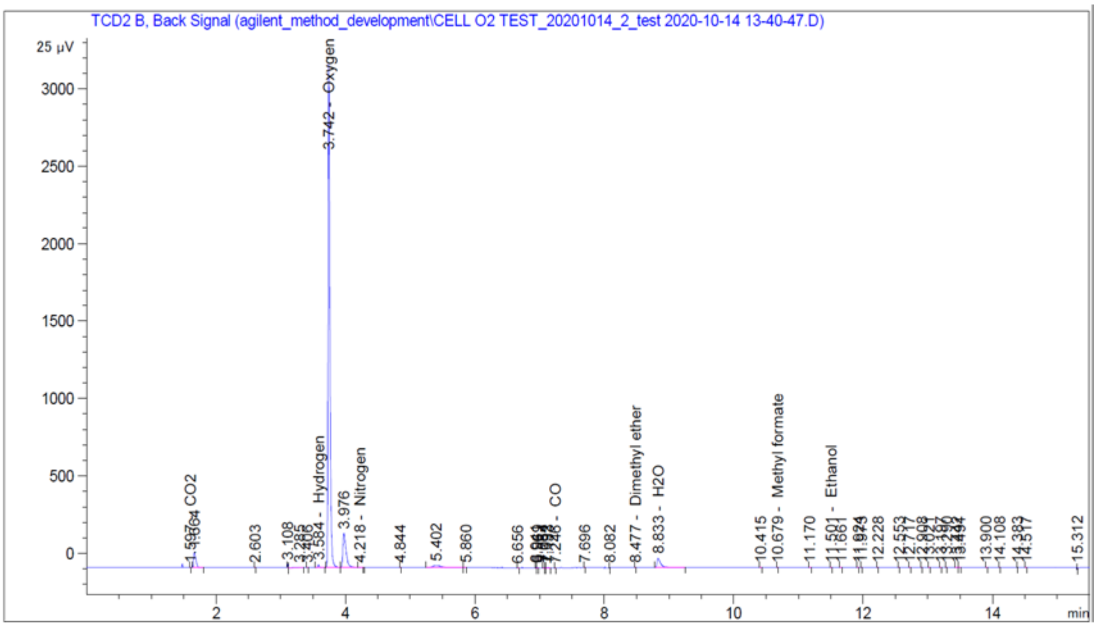

b

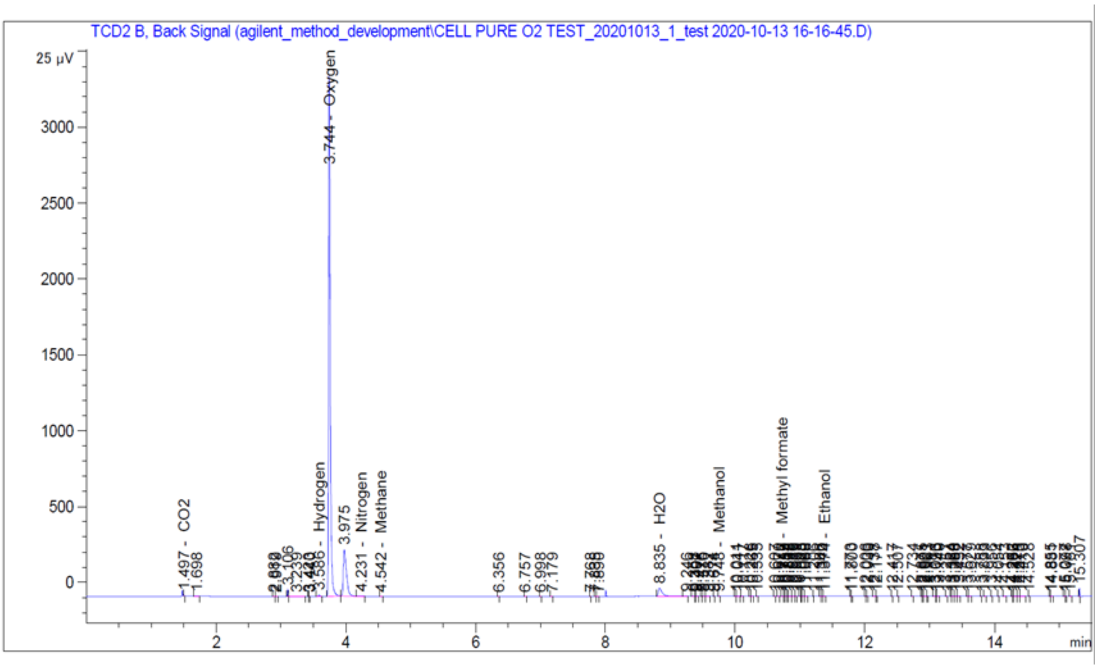

Supplementary Fig.21 Gas product from the anode. a Composition of the gas production at the anode by electrolyzing H_2SO_4 solution checked by gas chromatography (GC.). **b** Pure O_2 checked by GC.

Compared with Supplementary Fig.21a and Fig.21b, we can confirm that the gas production at the anode is high purity O_2(>99%).

15. DAE module operating condition with triple junction solar cell

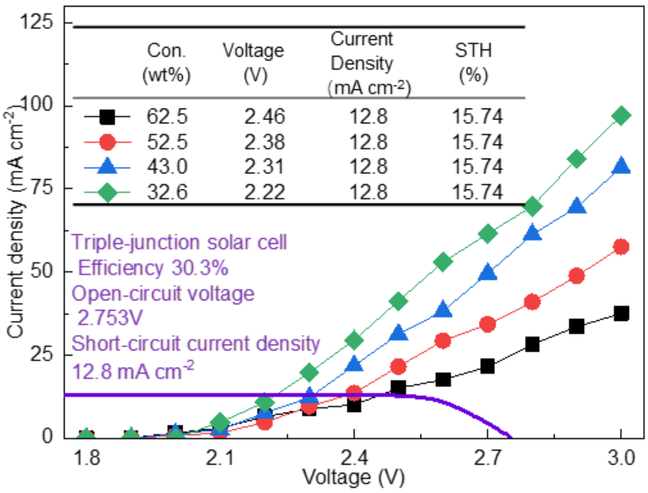

Supplementary Fig.22 The triple junction solar cell[7] and DAE module performance under different H_2SO_4 concentration. Source data are provided as a Source Data file.

The Solar to hydrogen efficiency (STH) is calculated by following:

$$\mathrm{STH}(\%)=\frac{1.23\mathrm{V}\times\text{Current Density}}{\text{Solar Intensity}}\times\eta_{f,H_2} \tag{1}$$

η_{f,H_2} represents the faradaic efficiency for hydrogen evolution, which is assumed to be 100%, and 1.23 V represents the thermodynamic potential for water splitting at room temperature[8]. Under each R.H., the current density keeps at12.8 mA cm^{-2}, so the STH(%) under all R.H. is:

$$\mathrm{STH}(\%)=\frac{1.23\ \mathrm{V}\times 12.8\ \mathrm{mA\ cm^{-2}}}{100\ \mathrm{mW\ \ cm^{-2}}}\times 100\%=15.74\% \tag{2}$$

16. DAE module driven by wind turbine

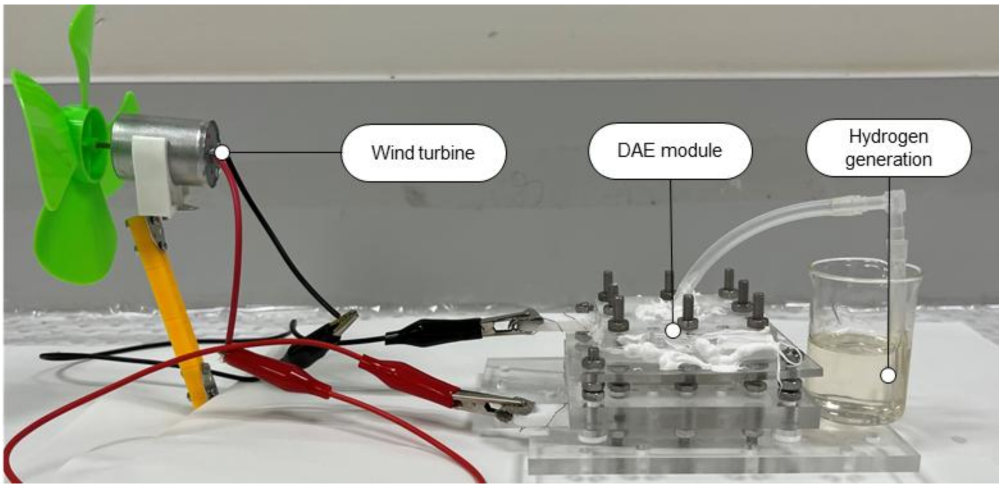

Supplementary Fig.23 A photo of the DAE module coupled with the wind turbine. Video demonstration can be found in Supplementary Movie 5.

17. Supplementary tables

Supplementary Table 1 Effects of R.H. on stable voltage, energy efficiency and STH efficiency under constant current density 15.0 mA cm^{-2} for 48 hours.

R.H. (%)	Voltage (V)	Energy efficiency (%)	STH efficiency (%)
20	2.81	43.8	20.6
40	2.53	48.6	22.9
60	2.40	51.3	24.2
80	2.33	52.8	24.9

The energy efficiency is calculated by following:

$$\text{Efficiency } \%=\frac{1.23\text{V}}{\text{Voltage}}\times\eta_{f,H_2} \quad (3)$$

η_{f,H_2} represents the faradaic efficiency for hydrogen evolution, which is assumed to be 100%, and 1.23 V represents the thermodynamic potential for water splitting at room temperature[8].The solar-to-hydrogen(STH) efficiency is calculated by following:

$$\text{STH}(\%)=\text{PV efficiency}\times\frac{1.23\text{V}}{\text{Voltage}}\times\eta_{f,H_2} \quad (4)$$

While the PV efficiency is assumed to be 47.1%, which is based on the multi-junction concentrator solar cell[9].

Supplementary Table 2 Performance of the hydrogen production unit of this work against references.

Ref.	High purity hydrogen **at output**	Free of inert gas carrier at cathode	Use of direct air	Free of Photo-catalyst	#Free of membrane	Suitable for (semi-)arid environment	Long-term Stability	Hydrogen production rate (L m^{-2} h^{-1}) Θ	Theoretical STH (%)
**Guo et al. (this work)**	**√ (>99%)**	√	√	√	√	√	√	**93.1**	**15.7-24.9**
10. Rongé et al. (2014)[Ⱶ]	×	×	√	×	×	×	√	0.025	0.0068
11. Xu et al. (2019)[*]	×	×	√ humidified	×	×	×	×	/	NA
12. Spurgeon et al. (2011)[T]	×	×	√ humidified	√	×	×	×	/	NA
13. Kumari et al. (2016)[§]	× (<2%)	×	√ humidified	√	×	×	√ high R.H. only	20.6	6.2
14. Zafeiropoulos et al.(2019)[¤]	× (<1%)	×	√ humidified	×	×	×	√	1.9	NA
15. Fornaciari et al. (2020)[φ]	×	×	×	√	×	×	×	/	NA
16. Kistler et al. (2020)[‡]	× (<2%)	×	×	√	×	×	√	63.1	14
17. Heremans et al. (2017)	× (<1%)	×	×	√	×	×	√	55.8	15.1
18. Heremans et al. (2019)	× (<1%)	×	×	√	×	×	√	45.7	NA
19. Amano et al. (2018)[ψ]	× (<1%)	×	×	×	×	×	√	0.59	NA
20. Amano et al. (2020)[Θ]	× (<2%)	×	×	×	×	×	√	7.3	NA
21. Chen et al. (2020)	×	NA	NA	×	√	×	×	/	NA
22. Daeneke et al. (2017)	×	NA	NA	×	√	×	√	1.6	0.44
23. Nishiyama et al. (2021)[Ø]	× (66%)	NA	NA	×	√	×	√	2.5	0.76

Use of ion exchange membrane inevitably mixes the product H_2 gas with the feed gas resulting in very low H_2 purity.

Θ Assume faradaic efficiency=100%.

Ⱶ The cathode compartment was flushed with dry nitrogen and sealed.

* Air was artificially humidified to 80% relative humidity.

Ƭ Only demonstrated high R.H. (95%) humidified air.

§ Only demonstrated high R.H. (80%) humidified air, with the hydrogen faradaic efficiency=63%.

¤ Air was artificially humidified to 60% R.H. at 30°C, or heat the electrolyzer to 50 °C and 70 °C for 30% R.H, and the PEC performance of the Ti/TiO_2 and W/WO_3 photoanodes was evaluated under LED-365 nm and LED-415 nm light illumination separately at 1.23 V vs RHE.

φ Electrolyzer was heated up to 80 °C for 30% R.H. humid argon was fed to both cathode and anode for reaching nearly 200 mA cm^{-2} at 2V operating potential.

‡ Extra heater was used to heat the bubble humidifier to 70 °C; N_2 carrier gas was humidified to near saturation through a 70 °C bubble humidifier before feeding into the anode, and dry N_2 was used for cathode; outdoor experiments lack details of feed gas composition.

ψ Electrolyzer was operated with an applied voltage of 1.2V at 453 nm light irradiation

Θ Electrolyzer was operated with an applied voltage of 1.2V at 365 nm UV irradiation

Ø Liquid water was used, and hydrogen and oxygen production mixed, so a gas separation facility was needed.

Supplementary Table 3 Comparison between other types of electrolyzers and our work.

	AWE[24-26]	PEMWE[24-26]	AEMWE[26]	**Our work-DAE(H_2SO_4)**	**This work-DAE(KOH)**
Pressure (Bar)	10-30	20-50	atm.	**atm.**	**atm.**
Temperature(°C)	60-90	50-80	N/A	**Room temperature**	**Room temperature**
Current Density (mA cm^{-2})	250-450	1000-2000	200–500	**10-200**	**10-574**
Cell Voltage (V)	1.8-2.4	1.8-2.2	N/A	**2.2-3.0**	**1.8-4.0**
Energy efficiency (%) (LHV)	51-68	56-68	N/A	**41-51**	**31-68**
Electrocatalyst cost	Low	High	Low	**High**	**Low**
Electrolyzer Cost*	High	Very High	Medium	**Low**	**Low**

* Use of pressure vessel significantly increase the electrolyzer cost.

Supplementary Table 4 Comparison between other two solar-driven water splitting systems and our work.

	Solar to Hydrogen efficiency (%)
PC [23]	~1
PEC (unassisted)[27,28]	~4
PEC (Photoelectrode coupled with PV devices) [29,30]	~8
**This work: PV-DAE (H_2SO_4)**	**11~15** (based on silicon heterojunction solar cell[31])<br>**19~26** (based on multi-junction concentrator solar cell[9])
**This work: PV-DAE (KOH)**	**8~18** (based on silicon heterojunction solar cell[31])<br>**14~32** (based on multi-junction concentrator solar cell[9])

18. Supplementary Note 1

In order to estimate how much air humidity is changed during the operation of a commercial scale DAE farm, we did the following calculations using Alice Spring where Uluru is nearby as an example:

Supplementary Table 5 The conditions in Alice Spring

	Value	Ref.	Notes
Thermodynamic potential for water splitting	1.23 V	8	
Temperature, T	27.8 °C	32	3pm
Relative humidity, R.H.	25%	32	3pm
Wind speed	15.0 km h^{-1}	32	3pm
Population	26534	33	2018
Area	327.7 km^2	33	
Thickness of ground air	30 m		
solar exposure daily	6.0 kwh m^{-2} day^{-1}	34	2020
Solar exposure hour	10 h day^{-1}	35	
Solar to electricity efficiency	20 %	36	
Electricity to H_2 efficiency	44 %		
Average power consumption	5662 kwh $person^{-1}$ $year^{-1}$	37	NSW

.

Annual solar intensity:

$$6.0\ \text{kwh m}^{-2}\ \text{day}^{-1} \times 365\text{day year}^{-1} = 2190\ \text{kwh m}^{-2}\ \text{year}^{-1} \tag{5}$$

Annual H_2 production from solar:

$$2190\ \text{kwh m}^{-2}\ \text{year}^{-1} \times 20\ \% \times 44\ \% = 192.720\ \text{kwh m}^{-2}\ \text{year}^{-1} \tag{6}$$

Annual overall energy consumption:

$$5662\ \text{kwh person}^{-1}\text{year}^{-1} \times 26534\ \text{person} = 1.502 \times 10^{8}\ \text{kwh year}^{-1} \tag{7}$$

Area for DAE farm:

$$1.502\times10^{8}\ \text{kwh year}^{-1}\div192.720\ \ \text{kwh m}^{-2}\ \text{year}^{-1}\div\ 10^{6}\ \text{m}^{2}\ \text{km}^{-2}=0.780\ \text{km}^{2} \quad (8)$$

Overall current driven by solar cell:

$$\frac{1.502\times10^{8}\ \text{kwh year}^{-1}\times\left(3.6\times10^{6}\right)\ \text{J kwh}^{-1}}{1.23\ \text{V}\times365\ \text{day year}^{-1}\ \times10\ \text{h day}^{-1}\times3600\ \text{s h}^{-1}}=3.346\times10^{7}\ \text{A} \quad (9)$$

Overall water consumption rate:

$$\frac{3.346\times10^{7}\times6.25\times10^{18}}{2\times6.02\times10^{23}}\ \text{mol s}^{-1}\times3600\ \text{s h}^{-1}\times18.\ 02\ \text{g mol}^{-1}=1.127\times10^{7}\ \text{g h}^{-1} \quad (10)$$

For T=27.8 °C, R.H.=25%, the absolute humidity is 6.72 g cm^{-3}.

Assume the overall volume of air is easy for mixing, and the area of the Alice spring is square, the side is:

$$\sqrt{327.7\ \text{km}^{2}}=18.102\ \text{km} \quad (11)$$

Hence, the mass flow rate for water carried by the air flow into the area is:

$$6.72\ \text{g m}^{-3}\times18.102\ \text{km}\times15.0\ \text{km h}^{-1}\times30\ \text{m}\times10^{6}\text{m}^{2}\ \text{km}^{-2}=5.474\times10^{10}\ \text{g h}^{-1} \quad (12)$$

The mass flow rate for water left in the area is Eq(12) – Eq(10):

$$1.825\times10^{10}\ \text{g h}^{-1}\text{-}1.127\times10^{7}\ \text{g h}^{-1}=1.824\times10^{10}\ \text{g h}^{-1} \quad (13)$$

The average R.H. of the air in this area after electrolysis is:

$$25\ \%\div\left(5.474\times10^{10}\ \text{g h}^{-1}\right)\times5.473\times10^{10}\ \text{g h}^{-1}=24.995\ \% \quad (14)$$

The R.H. of the air was changed from 25% to 24.995% in the presence of the DAE farm, which has negligible impact on the local environment. Also, only 0.780 km^2 is needed for building the DAE farm.

According to the literature[38], we recalculated the change of R.H. by using a surface layer of 20m your important comment. In comparison, we also include the results using a surface layer of 10 m. In the case of 30m, 20m, and 10m, the R.H. was reduced from 25% to 24.995%, 24.992%, and 24.98%, respectively.

Supplementary Reference

1 World Resources Institute (WRI) Aqueduct. *Aqueduct water risk atlas*, < aqueduct.wri.org> (2018).

2 World Bank Group. *Photovoltaic power potential*, <globalsolaratlas.info> (2019).

3 Technical University of Denmark (DTU). *Wind power density potential*, <globalwindatlas.info> (2017).

4 Darling, H. E. Conductivity of Sulfuric Acid Solutions. *J. Chem. Eng. Data* **9**, 421-426, (1964).

5 Muller, T. L. *Kirk-Othmer Encyclopedia of Chemical Technology*, *Sulfuric Acid and Sulfur Trioxide* (John Wiley & Sons, Inc., 2000).

6 Rhodes, F. & Barbour, C. The viscosities of mixtures of sulfuric acid and water. *Ind. Eng. Chem.* **15**, 850-852, (1923).

7 Yang, W.-x. *et al.* Investigation of room-temperature wafer bonded GaInP/GaAs/InGaAsP triple-junction solar cells. *Applied Surface Science* **389**, 673-678, (2016).

8 Nakamura, A. *et al.* A 24.4% solar to hydrogen energy conversion efficiency by combining concentrator photovoltaic modules and electrochemical cells. *Appl. Phys. Express* **8**, 107101, (2015).

9 Geisz, J. F. *et al.* Six-junction III–V solar cells with 47.1% conversion efficiency under 143 Suns concentration. *Nat. Energy* **5**, 326-335, (2020).

10 Rongé, J. *et al.* Air-based photoelectrochemical cell capturing water molecules from ambient air for hydrogen production. *RSC Adv.* **4**, 29286-29290, (2014).

11 Xu, K. *et al.* Hydrogen from wet air and sunlight in a tandem photoelectrochemical cell. *Int. J. Hydrog. Energy* **44**, 587-593, (2019).

12 Spurgeon, J. M. & Lewis, N. S. Proton exchange membrane electrolysis sustained by water vapor. *Energy Environ. Sci.* **4**, 2993-2998, (2011).

13 Kumari, S., Turner White, R., Kumar, B. & Spurgeon, J. M. Solar hydrogen production from seawater vapor electrolysis. *Energy Environ. Sci.* **9**, 1725-1733, (2016).

14 Zafeiropoulos, G., Johnson, H., Kinge, S., van de Sanden, M. C. M. & Tsampas, M. N. Solar Hydrogen Generation from Ambient Humidity Using Functionalized Porous Photoanodes. *ACS Appl. Mater. Interfaces* **11**, 41267-41280, (2019).

15 Fornaciari, J. C. *et al.* The Role of Water in Vapor-fed Proton-Exchange-Membrane Electrolysis. *J. Electrochem. Soc.* **167**, 104508, (2020).

16 Kistler, T. A., Um, M. Y. & Agbo, P. Stable Photoelectrochemical Hydrogen Evolution for 1000 h at 14% Efficiency in a Monolithic Vapor-fed Device. *J. Electrochem. Soc.* **167**, 066502, (2020).

17 Heremans, G. *et al.* Vapor-fed solar hydrogen production exceeding 15% efficiency using earth abundant catalysts and anion exchange membrane. *Sustain. Energy Fuels* **1**, 2061-2065, (2017).

18 Heremans, G., Bosserez, T., Martens, J. A. & Rongé, J. Stability of vapor phase water electrolysis cell with anion exchange membrane. *Catal. Today* **334**, 243-248, (2019).

19 Amano, F., Shintani, A., Mukohara, H., Hwang, Y.-M. & Tsurui, K. Photoelectrochemical Gas–Electrolyte–Solid Phase Boundary for Hydrogen Production From Water Vapor. *Front. Chem.* **6**, 598, (2018).

20 Amano, F. *et al.* Vapor-fed photoelectrolysis of water at 0.3 V using gas-diffusion photoanodes of SrTiO3 layers. *Sustain. Energy Fuels* **4**, 1443-1453, (2020).

21 Chen, Y. *et al.* Water collection from air by ionic liquids for efficient visible-light-driven hydrogen evolution by metal-free conjugated polymer photocatalysts. *Renew. Energy* **147**, 594-601, (2020).

22 Daeneke, T. *et al.* Surface Water Dependent Properties of Sulfur-Rich Molybdenum Sulfides: Electrolyteless Gas Phase Water Splitting. *ACS Nano* **11**, 6782-6794, (2017).

23 Nishiyama, H. *et al.* Photocatalytic solar hydrogen production from water on a 100 m2-scale. *Nature* **598**, 304–307, (2021).
24 Buttler, A. & Spliethoff, H. Current status of water electrolysis for energy storage, grid balancing and sector coupling via power-to-gas and power-to-liquids: A review. *Renew. Sust. Energ. Rev.* **82**, 2440-2454, (2018).
25 Carmo, M., Fritz, D. L., Mergel, J. & Stolten, D. A comprehensive review on PEM water electrolysis. *Int. J. Hydrog. Energy* **38**, 4901-4934, (2013).
26 Abbasi, R. *et al.* A Roadmap to Low-Cost Hydrogen with Hydroxide Exchange Membrane Electrolyzers. *Adv. Mater.* **31**, 1805876, (2019).
27 Pan, L. *et al.* Boosting the performance of Cu2O photocathodes for unassisted solar water splitting devices. *Nat. Catal.* **1**, 412-420, (2018).
28 Kobayashi, H. *et al.* Development of highly efficient CuIn0.5Ga0.5Se2-based photocathode and application to overall solar driven water splitting. *Energy Environ. Sci.* **11**, 3003-3009, (2018).
29 Kim, J. H. *et al.* Hetero-type dual photoanodes for unbiased solar water splitting with extended light harvesting. *Nat. Commun.* **7**, 1-9, (2016).
30 Wang, S., Liu, G. & Wang, L. Crystal Facet Engineering of Photoelectrodes for Photoelectrochemical Water Splitting. *Chem. Rev.* **119**, 5192-5247, (2019).
31 Yoshikawa, K. *et al.* Silicon heterojunction solar cell with interdigitated back contacts for a photoconversion efficiency over 26%. *Nat. Energy* **2**, 1-8, (2017).
32 Bureau of Meteorology. *Climate statistics for Australian locations-Alice Springs Airport*, <http://www.bom.gov.au/climate/averages/tables/cw_015590_All.shtml> (2021).
33 Australian Bureau of Statistics. 3218.0– *Regional Population Growth, Australia, 2017-18: Population Estimates by Local Government Area (ASGS 2018), 2017 to 2018*, <https://www.abs.gov.au/AUSSTATS/abs@.nsf/DetailsPage/3218.02017-18> (2019).
34 Bureau of Meteorology. *Monthly mean daily global solar exposure-Alice Springs Airport*, <http://www.bom.gov.au/jsp/ncc/cdio/weatherData/av?p_nccObsCode=203&p_display_type=dataFile&p_startYear=&p_c=&p_stn_num=015590> (2021).
35 Bureau of Meteorology. *Average annual & monthly sunshine duration*, <http://www.bom.gov.au/jsp/ncc/climate_averages/sunshine-hours/index.jsp> (2016).
36 Solar Calculator. *Solar panel efficiency*, <https://solarcalculator.com.au/solar-panel-efficiency/#:~:text=The%20average%20cell%20efficiency%20of,will%20produce%20per%20square%20metre.> (2021).
37 Frontier Economics Pty Ltd. *Residential energy consumption benchmarks*. (2020).
38 Wallace, J. M. & Hobbs, P. V. *Atmospheric science: an introductory survey* Vol. 92 (Elsevier, 2006).
