# Supplementary material for: Hydrogen production from the air
Source: Nat Commun. 2022 Sep 6;13:5046. doi: 10.1038/s41467-022-32652-y (PMC9448774; doi:10.1038/s41467-022-32652-y)
Supplement: Supplementary file 3 — Description of Additional Supplementary Files [file 41467_2022_32652_MOESM3_ESM.pdf]

Description of Additional Supplementary Files

File Name: Supplementary Movie 1
Description: Free movement of dyed electrolyte in the capillaries of glass foam.

File Name: Supplementary Movie 2
Description: Prototype of hydrogen generation tower.

File Name: Supplementary Movie 3
Description: Effect of solar intensity.

File Name: Supplementary Movie 4
Description: Real-time hydrogen generation by solar-driven DAE.

File Name: Supplementary Movie 5
Description: Real-time hydrogen generation by DAE coupled with wind turbine.
